# Tuberculosis Pericarditis with Cardiac Tamponade: Management in the Resource-Limited Setting

**DOI:** 10.4269/ajtmh.2010.10-0271

**Published:** 2010-12-06

**Authors:** Tom Heller, Richard J. Lessells, Claudia Wallrauch, Enrico Brunetti

**Affiliations:** Hlabisa Hospital, Hlabisa, KwaZulu-Natal, South Africa; Africa Centre for Health and Population Studies, University of KwaZulu-Natal, Mtubatuba, KwaZulu-Natal, South Africa; Division of Infectious and Tropical Diseases, University of Pavia, San Matteo Hospital Foundation, Pavia, Italy

## Abstract

We report a case of human immunodeficiency virus–associated pericardial tuberculosis complicated by cardiac tamponade. Emergency management and subsequent therapeutic interventions are described and then discussed with particular focus on resource-limited settings. The paucity of evidence to support clinical decisions is emphasized and the need for well designed diagnostic and therapeutic studies is highlighted.

The convergence of human immunodeficiency virus (HIV) and tuberculosis (TB) epidemics in southern Africa has contributed to a significant increase in reported cases of extrapulmonary TB (EPTB).^1^ One of the most common presentations of disease is pericardial TB, which in HIV co-infection is associated with significant mortality and morbidity.^2^ Cardiac tamponade is a common and life-threatening complication of TB pericarditis that requires immediate medical intervention.

We report a case of TB pericarditis associated with HIV infection and its management in Hlabisa Hospital, a rural district hospital in northern KwaZulu-Natal, South Africa. Hlabisa is located at the epicenter of the intertwined HIV and TB epidemics: the prevalence of HIV is 21.5% in the adult population (15–49 years of age); the TB notification rate was 1,700 per 100,000 persons in 2008; and the HIV co-infection rate for TB patients is 76%.^3^ We discuss the identification and management of this condition in HIV-infected persons, with a focus on settings with limited resources for diagnosis and intervention. We highlight some of the key areas where further evidence is required to inform clinical practice.

## Case Report

**Figure 1.**
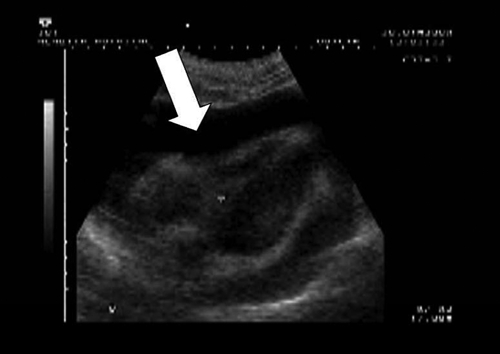
Ultrasound at presentation for the patient, showing large pericardial effusion and right ventricular collapse.

A 34-year old HIV-infected man (CD4 cell count = 310 cells/mm^3^) came to our hospital with a short history of increasing dyspnea and dizziness. He reported chest pain, weight loss, night sweats, but no cough. On initial assessment, he was unwell, diaphoretic, tachycardic (heart rate = 128/min), hypotensive (blood pressure = 100/60 mm of Hg), and severely tachypnoeic (respiratory rate = 40/min). Jugular venous pressure was increased. Chest radiograph demonstrated enlarged cardiac shadow. Because echocardiography was available on site, ultrasound was performed using a conventional B-Mode ultrasound scanner (Just Vision 400; Toshiba, Tokyo, Japan) with a 3.5-MHz abdominal probe and a sub-xiphoidal approach. A 3–4 cm pericardial effusion was seen, with impaired filling of the right ventricle and right atrium, the characteristic sonographic signs of cardiac tamponade (Figure 1 and Supplementary Video 1).

**Figure 2.**
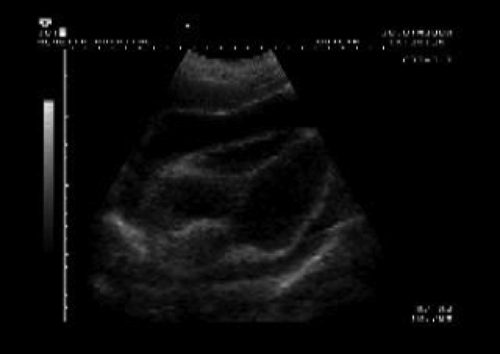
Ultrasound post-pericardiocentesis for the patient, showing persistent large effusion but improved right ventricular filling.

**Figure 3.**
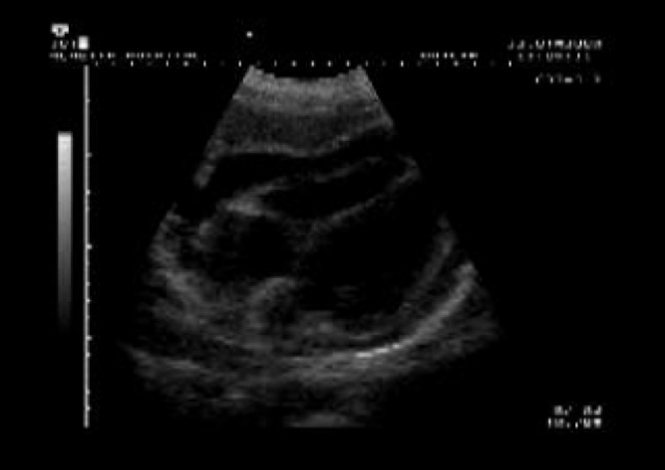
Ultrasound on day 3 for the patient, showing reduction in pericardial effusion.

The patient was transferred to the high care unit for pericardiocentesis and continuous electrocardiography (ECG) monitoring. No dedicated pericardiocentesis set or central venous cannula was available at that time. Localization for intercostal puncture approximately in the mid-clavicular line was determined by using ultrasound, and a 16-gauge intravenous cannula was inserted in the lower portion of the intercostal space. The needle was removed and 400 mL of clear yellow fluid was drained before removal. Despite the persistence of a large effusion, cardiac function improved significantly, and the clinical status improved significantly within thirty minutes (Figure 2 and Supplementary Video 2). Anti-tuberculous therapy (Rifafour^®^; Sanofi-Aventis, Midrand, South Africa, four tablets/day, a fixed-dose combination of rifampicin, isoniazid, ethambutol, and pyrazinamide)) and treatment with prednisolone (80 mg/day) were commenced. After two days, the condition had stabilized and the patient requested discharge; at this time the effusion measured 1–2 cm (Figure 3). Prednisolone was prescribed for one month and continued TB therapy was supervised at the primary health care clinic. After two months, antiretroviral therapy (stavudine/lamivudine/efavirenz) was initiated. The six-month course of antituberculous therapy was completed uneventfully with no evidence of immune reconstitution inflammatory syndrome and ultrasound at the end of treatment demonstrated no residual pericardial effusion.

## Discussion

Clinical manifestations of pericarditis (chest pain, cough, dyspnea) overlap with other disease entities in HIV-infected persons: most importantly pulmonary and pleural TB, but also bacterial pneumonia and *Pneumocystis jiroveci* pneumonia. Systemic signs (weight loss, night sweats, fatigue) may be present but are non-discriminatory. Signs of heart failure may result from progression of pericardial effusion but HIV-infected persons are also more likely to have myocardial involvement and hemodynamic instability.^4^

A high index of suspicion for pericardial disease is therefore required in symptomatic HIV-infected persons, and it is important that imaging is included in algorithms for smear-negative and extrapulmonary TB.^5^ Although expansion of radiographic facilities would be welcomed, we would also like to see more extensive use of ultrasound techniques. Although cardiomegaly observed by chest radiograph is suggestive of pericardial effusion, distinction from dilated cardiomyopathy requires ultrasound and formal echocardiography is rarely available in the district hospital setting. Recognition of the anechoic effusion is normally straightforward; thickened pericardium with echogenic, exudative coating and fibrin strands may be seen and increases the likelihood of tuberculous etiology.^6^ If expertise is available, it is also important to assess the right ventricular cavity because in cases of tamponade the diameter will be reduced and there might be a paradoxical inward motion or even collapse of the free wall.

Preliminary work from our group suggests that non-specialist physicians can be trained to identify key features of EPTB by using ultrasound.^7^ However, pericardial effusion was a common finding in HIV-infected patients in this study and further research is required to establish clinical decision–making tools based on clinical features and sonographic appearance.

In countries with a high prevalence of TB, this disease has been reported as the cause of more than 90% of large pericardial effusions in HIV-infected persons.^8^ Other potential causes include bacterial pericarditis (e.g., non-typhoidal *Salmonella*), human herpesvirus-8–related disease, and lymphoma. Definitive diagnosis of tuberculous etiology is challenging because studies have shown the yield from direct smear examination of pericardial fluid to be as low as 2%^9^; culture of pericardial fluid is positive in 38–56% of cases.^9^,^10^ Other indirect methods used to support TB diagnosis, such as adenosine deaminase levels in pericardial fluid, have the limitation of lower sensitivity in persons with advanced HIV infection.^11^ All diagnostic studies have the common problem of the lack of a definitive gold standard. Whether novel diagnostic technologies that use the polymerase chain reaction have sufficient accuracy with samples such as pericardial fluid requires well-designed studies.

In the absence of sufficiently rapid, accurate diagnostic techniques, it is appropriate in high prevalence areas to initiate empiric antituberculous therapy in the presence of large pericardial effusion. Diagnostic pericardial aspiration should be reserved for cases where there is evidence for an alternative diagnosis (e.g., skin lesions of Kaposi's sarcoma) or suspicion of drug-resistant TB.

Pericardiocentesis is often a life-saving intervention and is essential in patients with cardiac tamponade. In clinical studies, the proportion of patients with tamponade has ranged between 20% and 90% depending on the selection of patients and definition of tamponade.^4^,^12^ Cardiac tamponade is classically a clinical diagnosis characterized by increased jugular venous pressure, hypotension, and diminished heart sounds but cardiac ultrasound may identify typical features such as collapse of the right ventricle or right atrium.

**Figure 4.**
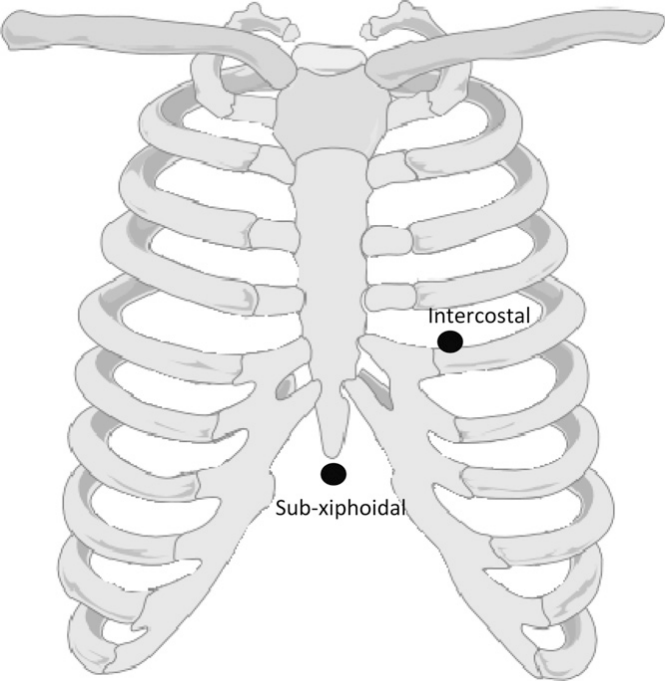
Sites for pericardiocentesis. **A**, Standard sub-xiphoidal approach. **B**, Intercostal approach used with ultrasound guidance in this case.

According to standard protocols, a needle attached to the V_1_ lead of an electrocardiograph machine is inserted into the pericardial space, commonly by a sub-xiphoidal approach. Echocardiographic control is recommended but the expertise is unlikely to be available in many settings. In settings where dedicated pericardiocentesis equipment is not available, the use of alternative approaches, such as intercostal approach with a simple cannula as described in our case, might be necessary but should be guided by ultrasound (Figure 4). It is necessary to drain only enough fluid to relieve the signs of hemodynamic instability. Many patients experience rapid clinical improvement despite the continuing presence of pericardial effusion. It is worth remembering that the aim is reduction of pressure in the pericardial space not volume *per se* and the drainage of 50–100 mL of fluid might suffice for resolution of tamponade.

Antituberculous therapy should be commenced as soon as a diagnosis of pericarditis is made in a setting with high prevalence of TB and HIV infection. Treatment regimens recommended for pericardial TB are the same as for pulmonary TB, consisting of rifampicin, isoniazid, ethambutol, and pyrazinamide for two months, followed by rifampicin and isoniazid for four months.^13^ The paucity of data informing the treatment of TB/HIV co-infection has recently been highlighted, with particular uncertainty for duration of therapy.^14^ This is particularly striking for EPTB and it is crucial that common forms of EPTB are adequately represented in future trials of antituberculous therapy.

Corticosteroids are frequently prescribed for TB pericarditis but the evidence to support their use in HIV-infected patients is limited. Studies conducted in the pre-HIV era suggested significant reduction in mortality with steroids in pericardial effusion and constrictive pericarditis.^15^,^16^ However, HIV infection influences the pathologic process and evidence suggests that constrictive pericarditis is much less common. The only randomized trial in HIV-infected patients included only 58 patients and showed a non-significant trend to lower mortality with adjunctive steroids.^10^ A Cochrane review on this topic concluded that prednisolone has no proven beneficial effect in patients with TB pericarditis.^17^ An ongoing multicentre randomized trial aims to address this key question.^18^

It is important to remember that because rifampicin increases corticosteroid metabolism through hepatic enzyme induction, higher doses might be required.^19^ Prednisolone doses as high as 120 mg have been used successfully in treating patients with TB pericarditis.^20^

Pericardial TB is a World Health Organization stage IV condition and thus antiretroviral therapy is indicated regardless of CD4+ lymphocyte count.^21^ There is recent evidence to support the early initiation of combination antiretroviral therapy (ART) during antituberculous therapy for pulmonary TB, although the optimal time to start has yet to be determined.^22^ Patients with EPTB were not included in this trial and only observational data including patients with EPTB support the benefit of early antiretroviral therapy in this context.^23^

The potential risks of early ART initiation include drug toxicity, drug interactions, and immune reconstitution inflammatory syndrome. The occurrence of pericardial TB–immune reconstitution inflammatory syndrome has been reported but the morbidity or mortality associated with this is unknown.^24^ Theoretically, immune reconstitution could lead to re-accumulation of fluid (with the possibility of tamponade) and/or a higher risk of constrictive pericarditis. Our practice is to initiate ART after 2–8 weeks of TB treatment and, as far as possible, to monitor the clinical status closely.

## Conclusions

Pericardial TB is a frequent manifestation of HIV-associated TB and is associated with considerable mortality and morbidity. Many questions remain in terms of optimal diagnosis and therapy for this condition. As we progress into a new era of TB diagnostics and therapeutics to address this public health emergency, it is imperative that clinical trials include patients with extrapulmonary TB to answer some of the questions highlighted here.

## Supplementary Material

Supplementary Videos

## Supplementary Material

Supplementary Videos

## Supplementary Material

Supplementary Videos
