# Supplementary material for: Tuberculosis Pericarditis with Cardiac Tamponade: Management in the Resource-Limited Setting
Source: Am J Trop Med Hyg. 2010 Dec 6;83(6):1311–4. doi: 10.4269/ajtmh.2010.10-0271 (PMC2990051; doi:10.4269/ajtmh.2010.10-0271)
Supplement: Supplementary Videos [file SD6.pdf]

Supplementary Video 1. Video for the patient showing large pericardial effusion and right ventricular collapse characteristic of cardiac tamponade.

Supplementary Video 2. Video for the patient post-pericardiocentesis showing improved right ventricular filling.
